# Parasitic Contamination of Soil in the Southern United States

**DOI:** 10.4269/ajtmh.24-0075

**Published:** 2024-07-23

**Authors:** Christine Crudo Blackburn, Sally Mingshuang Yan, David McCormick, Lauren Nicholas Herrera, Roumen Borilov Iordanov, Mark Daniel Bailey, Maria Elena Bottazzi, Peter J. Hotez, Rojelio Mejia

**Affiliations:** ^1^ Department of Health Policy and Management, School of Public Health, Texas A&M University, College Station, Texas;; ^2^ Department of Pediatrics, National School of Tropical Medicine, Baylor College of Medicine, Houston, Texas

## Abstract

Parasites are generally associated with lower income countries in tropical and subtropical areas. Still, they are also prevalent in low-income communities in the southern United States. Studies characterizing the epidemiology of parasites in the United States are limited, resulting in little comprehensive understanding of the problem. This study investigated the environmental contamination of parasites in the southern United States by determining each parasite’s contamination rate and burden in five low-income communities. A total of 499 soil samples of approximately 50 g were collected from public parks and private residences in Alabama, Louisiana, Mississippi, South Carolina, and Texas. A technique using parasite floatation, filtration, and bead-beating was applied to dirt samples to concentrate and extract parasite DNA from samples and detected via multiparallel quantitative polymerase chain reaction (qPCR). qPCR detected total sample contamination of *Blastocystis* spp. (19.03%), *Toxocara cati* (6.01%), *Toxocara canis* (3.61%), *Strongyloides stercoralis* (2.00%), *Trichuris trichiura* (1.80%), *Ancylostoma duodenale* (1.42%), *Giardia intestinalis* (1.40%), *Cryptosporidium* spp. (1.01%), *Entamoeba histolytica* (0.20%), and *Necator americanus* (0.20%). The remaining samples had no parasitic contamination. Overall parasite contamination rates varied significantly between communities: western Mississippi (46.88%), southwestern Alabama (39.62%), northeastern Louisiana (27.93%), southwestern South Carolina (27.93%), and south Texas (6.93%) (*P* <0.0001). *T. cati* DNA burdens were more significant in communities with higher poverty rates, including northeastern Louisiana (50.57%) and western Mississippi (49.60%) compared with southwestern Alabama (30.05%) and southwestern South Carolina (25.01%) (*P* = 0.0011). This study demonstrates the environmental contamination of parasites and their relationship with high poverty rates in communities in the southern United States.

## INTRODUCTION

Neglected infections of poverty are infectious diseases that disproportionately affect marginalized communities.^1^ Many parasites are among these neglected infections of poverty, specifically *Ascaris lumbricoides*, *Cryptosporidium* spp. *Entamoeba histolytica*, *Giardia intestinalis*, *Strongyloides stercoralis*.^1^ The symptoms caused by these parasites vary but generally include diarrhea, anemia, and malnutrition.^2^ As a result, they can cause delays in cognitive and physical development in childhood, reinforcing the cycle of poverty.^3^^,^^4^

Previous work suggests that numerous parasitic diseases may be present in humans, particularly among people of color, in the Mississippi Delta, Cotton Belt, and Border regions.^1^ It has been estimated that there are millions of cases of ascariasis and toxocariasis in the southern United States alone.^5^^–^^7^ Studies have consistently shown that although parasitic infections are no longer widespread, they persist in vulnerable, high-poverty populations in the U.S. south and Appalachia regions.^8^

Despite their clinical and societal significance, there are limited studies to characterize the epidemiology of these parasites in the United States. Most systematic, high-quality studies of their prevalence were conducted from 1942 to 1982, meaning current information is limited.^7^ More recently, the 1999–2004 and 2009–2010 National Health and Nutrition Examination Survey (NHANES) analyzed the prevalence of some parasites, including *Toxocara canis* and *Toxocara cati*.^9^ Other recent studies characterized the population prevalence of several parasites in rural Alabama, peri-urban Texas, and among Latin American immigrants in Washington, DC.^2^^,^^10^^,^^11^

Environmental sampling provides an opportunity for broader epidemiological studies to address the limited existing data. This approach may indicate population-level prevalence and potential for transmission and thus demonstrate a need for further study and funding. Most environmental studies of parasites are wastewater-based epidemiology studies of protozoa, such as *Cryptosporidium* spp. and *G. intestinalis*.^12^^,^^13^ Such studies largely exclude helminths, primarily transmitted through the soil and whose life cycles entail defecation into the soil by humans or animals, followed by ingestion or dermal penetration from the soil by humans.^2^ Additionally, protozoa and heterokonts are also present in the soil and could therefore be transmitted through it.^14^ Some studies have aimed to detect *Toxocara* spp. and other soil-transmitted helminths in soil samples^15^^,^^16^; however, the two studies conducted in the United States sampled sewage sludge rather than soil.^15^^,^^17^ Furthermore, these studies used conventional microscopy-based detection of helminth eggs, which is subjective and inaccurate. In contrast, quantitative polymerase chain reaction (qPCR)-based molecular detection methods are more sensitive/specific, less labor intensive, and less time-consuming.^18^

**Figure 1.**
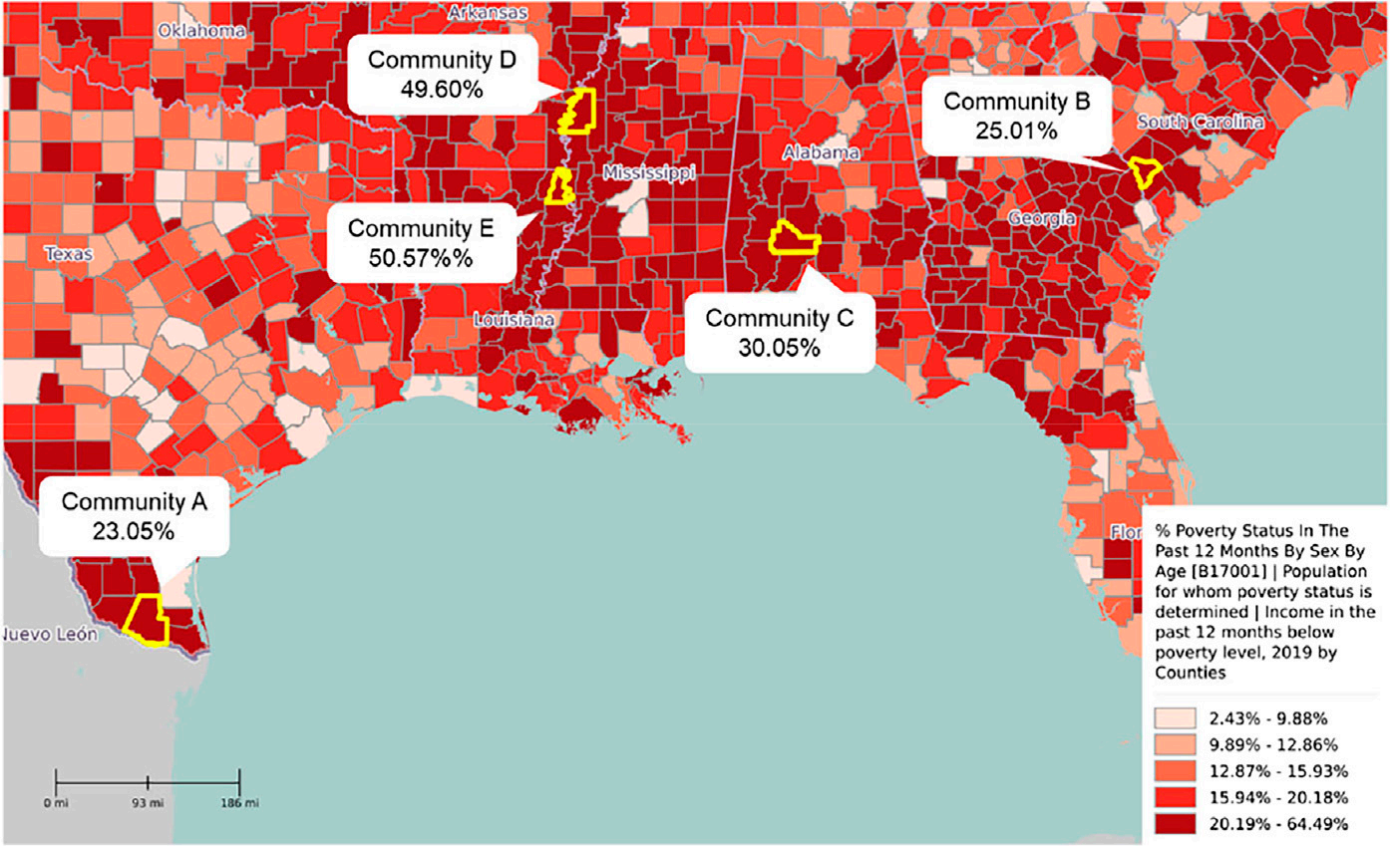
Map of communities and their respective poverty rates. Quintiles of poverty rates were retrieved from the 2019 American Community Survey and displayed around the time of soil sample collections.

This work involves the molecular detection of 11 parasites in soil samples in five low-income communities in the southern United States. The five communities are in the highest quintile for poverty rate in the United States and include cities and counties in southwestern South Carolina, northeastern Louisiana, south Texas, western Mississippi, and southwestern Alabama. This study determines the contamination rate and burden of each parasite in each community as possible indicators of the prevalence among human populations and the potential for endemic transmission in the United States. Furthermore, it examines the association between parasite contamination rates and poverty in the United States (Figure 1).

## MATERIALS AND METHODS

### Study design and sample collection.

Five communities in five states in the southern United States were selected as study communities. These included a county in southwestern South Carolina, a community in northeastern Louisiana, a city in south Texas, a community in western Mississippi, and a county in southwestern Alabama. Criteria for selecting communities included high poverty rate, low household median income, and rural status (Table 1). Only the community located in south Texas is classified as urban, and the decision to include this community was based on its proximity to large agricultural production and the U.S.–Mexico border. Approximately 100 samples from over four to six sites were collected from each community. Collection sites in each community were a combination of public parks and private residences. Sample locations at private homes were selected to represent the community accurately. Private residence sampling represented mostly low-income residences, as all communities sampled had high poverty rates. All low-income residences sampled were located along a road or street with other homes in town. Higher income residences sampled, however, were located outside of town, often on several acres of land with no additional houses in the proximity. Sampling of public spaces was conducted exclusively at high-traffic public areas where community members are most likely to come into contact with the soil, such as a public park.

**Table 1:** Communities selected for the study with their corresponding poverty rates (%), annual household median incomes ($), and populations (no. of people)*

Community	Location	Median Household Income ($)	Poverty Status (%)	Population	Sample Collection
A	South TX	$47,279	23.05	141,968	February 2019
B	Southwestern SC	$24,560	25.01	9,024	March 2019
C	Southwestern AL	$27,237	30.05	10,681	June 2019
D	Western MS	$20,857	49.60	2,004	December 2018
E	Northeastern LA	$17,801	50.57	2,753	October 2018

* Poverty rates, household median incomes, and populations were retrieved from the 2019 American Community Survey conducted by the U.S. Census Bureau. The following are the fully unabbreviated names of the community locations: south Texas (south TX), southwestern South Carolina (southwestern SC), southwestern Alabama (southwestern AL), western Mississippi (western MS), and northeastern Louisiana (northeastern LA).

Soil was collected for each sample by scraping a 50-mL conical centrifuge tube along the soil’s surface in different locations within the site. Samples were collected from October 2018 to June 2019. These samples were transported and stored at 4°C in the Laboratory of Human Parasitology, Baylor College of Medicine, Houston, Texas.

### Parasite floatation and filtration.

A DNA concentration technique used parasite flotation and filtration to concentrate parasite DNA from soil samples before DNA extraction. The mass of the samples—ranging from approximately 5 to 80 g—was determined and recorded before extraction. If more than 80 g of soil were collected for a sample, 50 g was used for parasite floatation and filtration, followed by DNA extraction, and the remainder was reserved. This technique was developed and optimized from a previous indoor dust study.^19^ In developing the parasite DNA extraction from dirt samples, briefly, 50 g of dirt was tested negative by multiparallel qPCR for all 11 parasites in this study. This standardized dirt was then spiked with eggs/larvae/cysts and serially diluted to detect 1 to 10 organisms per pathogen (results not shown).

Each sample was divided in half between two 50-mL conical centrifuge tubes. Phosphate-buffered saline (Alfa Asesar, Ward Hill, MA) with 0.05% TWEEN (Sigma-Aldrich, St. Louis, MO) was added to the 50 mL for each sample to wash macro-scale debris from the soil samples. The samples were vortexed for 5 minutes, centrifuged at 500 × *g* for 5 minutes, and the supernatant containing the debris was discarded.

To float helminth eggs and larva, as well as protozoa, 10 mL of a 35.6% NaNO_3_ solution (Vedco, St. Joseph, MO) with a specific gravity of 1.25 to 1.30 was added to the pellet in each conical centrifuge tube. The solution was vortexed for 5 minutes and centrifuged for 5 minutes at 500 × *g*.

The supernatant for each sample containing the floated parasites was then transferred to a filtration apparatus. The filtration apparatus consisted of a 60-mL syringe attached to a 50-mm syringe filter containing a nitrocellulose filter with 3 µm pores (Millipore Sigma, Burlington, MA), which is small enough to retain all parasites subsequently tested for. The filtration apparatus was attached to a vacuum manifold, which was, in turn, attached to a two-stage rotary vane vacuum pump (ELITech, Puteaux, France). Filtration with a vacuum pressure of as low as 25 µm Hg was performed until the eluent had passed through the filter.

### DNA extraction.

The MP Fast SpinKit for Soil (MP Biomedicals, Santa Ana, CA) was used with a modified protocol to extract DNA from parasites on the nitrocellulose filter. The modifications entailed preliminary steps to lyse parasite eggs. In brief, filters were transferred with tongue blades to a lysing solution. The solution contained a lysing matrix with ceramic, glass, and silica beads; 978 µL sodium phosphate buffer; 122 µL MT buffer; and an internal control DNA sequence subsequently used to confirm successful extraction.^20^ Heat disruption at 90°C for 10 minutes in a dry bath incubator followed by mechanical disruption by bead beating in the MP FastPrep 34-5G disruptor (MP Biomedicals, Santa Ana, CA) on speed 6 for 40 seconds was used to break open parasite eggs and lyse cells. Subsequent steps followed the standard protocol of the MP Fast SpinKit for Soil to extract DNA.^21^

### Quantitative polymerase chain reaction testing for parasite DNA.

The DNA extracted from samples was tested for *Ascaris lumbricoides*, *Ancylostoma duodenale*, *T. cati*, *T. canis*, *Cryptosporidium* spp., *Entamoeba histolytica*, *G. intestinalis*, *N. americanus*, *S. stercoralis*, *Trichuris trichiura*, and *Blastocystis* subtypes using multiparallel real-time qPCR.

To test for each parasite, a 7-µL reaction mixture was prepared for each sample. The reaction mixture consisted of 5 µL TaqMan^®^ Fast Advanced Master Mix (Applied Biosystems, Foster City, CA) with previously published forward primers (900 nM final concentration; Applied Biosystems), reverse primers (900 nM final concentration; Applied Biosystems), and FAM probe with a minor groove binder and nonfluorescent quencher (100 nM final concentration; Applied Biosystems) for each parasite (Table 2). Additionally, 2 µL of extracted DNA was added to each reaction mixture.^21^

**Table 2:** Target regions, primer sequences, and probe sequences by parasites for DNA amplification

Parasite	Target region	Forward primer sequence (5′ to 3′)
		Reverse primer sequence (5′ to 3′)
		Probe sequence (5′FAM to 3′)
*Ancylostoma duodenale*	ITS-2	GAATGACAGCAAACTCGTTGTTG ATACTAGCCACTGCCGAAACGT ATCGTTTACCGACTTTAG
*Ascaris lumbricoides*	ITS-1	TGCACATAAGTACTATTTGCGCGTAT CCGCCGACTGCTATTACATCA GAGCCACATAGTAAATT
*Cryptosporidium* spp.	DNA-J like protein	AACTTCACGTGTGTTTGCCAAT CCAATCACAGAATCATCAGAATCG CATATGAAGTTATAGGGATACCAG
*Blastocystis* spp.	16 s rRNA	AGTAGTCATACGCTCGTCTCAAA TCTTCGTTACCCGTTACTGC CGTGTAAATCTTACCATTTAGAGGA
*Entamoeba histolytica*	18 S rRNA	GTTTGTATTAGTACAAAATGGCCAATTC TCGTGGCATCCTAACTCACTTAGA CAATGAATTGAGAAATGACA
*Giardia intestinalis*	16 S rRNA	CATGCATGCCCGCTCA AGCGGTGTCCGGCTAGC AGGACAACGGTTGCAC
*Necator americanus*	ITS-2	CTGTTTGTCGAACGGTACTTGC ATAACAGCGTGCACATGTTGC CTGTACTACGCATTGTATAC
*Strongyloides stercoralis*	18 s rRNA	GAATTCCAAGTAAACGTAAGTCATTAGC TGCCTCTGGATATTGCTCAGTTC ACACACCGGCCGTCGCTGC
*Toxocara canis*	ITS-2	GCGCCAATTTATGGAATGTGAT GAGCAAACGACAGCSATTTCTT CCATTACCACACCAGCATAGCTCACCGA
*Toxocara cati*	ITS-2	ACGCGTACGTATGGAATGTGCT GAGCAAACGACAGCSATTTCTT TCTTTCGCAACGTGCATTCGGTGA
*Trichuris trichiura*	ITS-1	TCCGAACGGCGGATCA CTCGAGTGTCACGTCGTCCTT TTGGCTCGTAGGTCGTT

ITS = internal transcribed spacer; rRNA = ribosomal RNA.

A parasite-plasmid standard curve of 10-fold dilutions was generated for each parasite to serve as a positive control and to allow the quantification of the concentration of parasite DNA. Nuclease-free water was used as a negative control.^21^

The Fast Chemistry protocol for a 7-µL reaction volume was performed on the ABI Vii-A7, QuantStudio™ 3, or QuantStudio™ 7 Real-Time PCR systems with a hold stage and 40 cycles of amplification (Applied Biosystems) (Table 3). Results were analyzed on the QuantStudio™ Design and Analysis v2.6.0 software. On the basis of a previously established dynamic range using parasite-plasmid standards, samples were considered positive for cycle threshold <40.^22^

**Table 3:** Run method for fast chemistry protocol on real-time polymerase chain reaction systems

Stage		Temperature (°C)	Time (s)
Hold		95°C	20
Amplification	Denaturation	95°C	1
	Annealing and Extension	60°C	20

## STATISTICAL ANALYSES

The contamination rate and median parasite burdens were noted for each parasite in each community, and socioeconomic indicators were recorded. The contamination rate was calculated as the percentage of positive samples per the total number of samples tested. The parasite burden was defined as the concentration of the target DNA sequence for each parasite in fg/µL quantified using the standard curve and normalized by soil sample mass. Socioeconomic indicators for each community, including poverty rate, median household income, and GDP per capita, were obtained from the 2019 American Community Survey conducted by the U.S. Census Bureau.

Using these variables, statistical analysis was performed on GraphPad Prism v9.4.0 (GraphPad Software, La Jolla, CA). Specifically, χ^2^ tests were conducted to examine the association of socioeconomic indicators of communities with their parasite contamination rates. Kruskal–Wallis tests were used to investigate the association of socioeconomic indicators of communities with their median parasite burdens. Spearman’s rank correlation tests were applied to investigate the correlation between socioeconomic indicators of communities with parasite contamination rates or median parasite burdens.

## RESULTS

### Environmental contamination of parasites.

Four hundred ninety-nine samples were tested for *Ascaris lumbricoides*, *A. duodenale*, *Cryptosporidium* spp., *Entamoeba histolytica*, *G. intestinalis*, *N. americanus*, *S. stercoralis*, *T. canis*, *T. cati*, *T. trichiura*, and *Blastocystis* spp. through qPCR. The findings are summarized in Table 4. For the cohort, *Blastocystis* spp. was the parasite with the highest environmental contamination rate (19.03%, 95/499). Community D in western Mississippi had the highest contamination rate for *Blastocystis* spp. among the communities. (28.12%, 27/96), followed by Community C in southwestern Alabama (23.58%, 25/106), Community B in southwestern South Carolina (22.52%, 25/111), Community E in northeastern Louisiana (14.11%, 12/85), and Community D south Texas (5.94%, 6/101).

**Table 4:** Contamination rates by samples and sites for each parasite were detected overall and by the community*

Parasite	Contamination Rate by Samples	Contamination Rates by Sites
*Blastocystis* spp.		
Overall	19.03% (95/499)	72.97% (27/37)
Southwestern SC	22.52% (25/111)	100% (5/5)
Northeastern LA	14.11% (12/85)	55.56% (5/9)
South TX	5.94% (6/101)	57.14% (4/7)
Western MS	28.12% (27/96)	72.72% (8/11)
Southwestern AL	23.58% (25/106)	100% (5/5)
*Toxocara cati*		
Overall	6.01 (30/499)	37.83% (14/37)
Southwestern SC	0.90% (1/111)	20% (1/5)
Northeastern LA	9.41% (8/85)	44.44% (4/9)
Western MS	10.41% (10/96)	45.45% (5/11)
Southwestern AL	10.37% (11/106)	80% (4/5)
*Toxocara canis*		
Overall	3.61% (18/499)	24.32% (9/37)
Southwestern SC	2.70% (3/111)	40% (2/5)
Northeastern LA	2.35% (2/85)	22.22% (2/9)
Western MS	9.37% (9/96)	36.36% (4/11)
Southwestern AL	3.77% (4/106)	20% (1/5)
*Strongyloides stercoralis*		
Overall	2.00% (10/499)	13.51% (5/37)
Southwestern SC	5.40% (6/111)	40% (2/5)
Western MS	3.12% (3/96)	18.18% (2/11)
Southwestern AL	0.94% (1/106)	20% (1/5)
*Trichuris trichiura*		
Overall	1.80% (9/499)	13.51% (5/37)
Northeastern LA	1.18% (1/85)	11.11% (1/9)
Western MS	6.25% (6/96)	27.27% (3/11)
Southwestern AL	1.89% (2/106)	20% (1/5)
*Ancylostoma duodenale*		
Overall	1.42% (7/494)	13.51% (5/37)
Southwestern SC	0.91% (1/110)	20% (1/5)
Northeastern LA	2.38% (2/84)	22.22% (2/9)
South TX	0.99% (1/101)	14.28% (1/7)
Western, MS	3.12% (3/96)	9.09% (1/11)
*Giardia intestinalis*		
Overall	1.40% (7/499)	10.81% (4/37)
Southwestern SC	1.82% (2/110)	40% (2/5)
Western MS	4.17% (4/96)	9.09% (1/11)
Southwestern AL	0.94% (1/106)	20% (1/5)
*Cryptosporidium* spp.		
Overall	1.01% (5/493)	8.11% (3/37)
South TX	0.99% (1/101)	14.28% (1/7)
Southwestern AL	3.92% (4/102)	40% (2/5)
*Entamoeba histolytica*		
Overall	0.20% (1/497)	2.70% (1/37)
Southwestern AL	0.95% (1/105)	20% (1/5)
*Necator americanus*		
Overall	0.20% (1/499)	2.70% (1/37)
Western MS	1.04% (1/96)	0.91% (1/11)

* Contamination rate by sample is calculated as the percentage of positive samples per all samples tested for a parasite. Positive samples/all samples tested are displayed in parentheses. The contamination rate by sample is calculated as the percentage of sites with positive samples per all sites sampled in the geographic location of the study. Positive sites/all sites are displayed in parentheses. A site with one positive sample is considered positive for the parasite.

The zoonotic soil-transmitted helminths *T. cati* (6.01%, 30/499) and *T. canis* (3.61%, 18/499) had the next highest contamination rates. For *T. cati*, the contamination rates for the different communities in which *T. cati* was detected respectively were Community D in western Mississippi (10.41%, 10/96), Community C in southwestern Alabama (10.37%, 11/106), Community E in northeastern Louisiana (9.41%, 8/85), and southwestern South Carolina (0.90%, 1/111). Soil samples positive for *T. canis* were found in Community D in western Mississippi (9.37%, 9/96), Community C in southwestern Alabama (3.77%, 4/106), Community B in southwestern South Carolina (2.70%, 3/111), and Community A northeastern Louisiana (2.35%, 2/85).

Other soil-transmitted helminths detected in the soil included *S. stercoralis* (2.00%, 10/499), *T. trichiura* (1.80%, 9/499), *A. duodenale* (1.42%, 7/499), and *N. americanus* (0.20%, 1/499). Six of the nine positive samples for *T. trichiura* were collected from the community in western Mississippi (6.25%, 6/96). *N. americanus* was only detected in the community in western Mississippi (1.04%, 1/96), whereas *S. stercoralis* was found most commonly in southwestern South Carolina (5.40%, 6/111). No *Ascaris lumbricoides* were detected in these samples.

The protozoa *G. intestinalis *(1.40%, 7/499), *Entamoeba histolytica* (0.20%, 1/497), and *Cryptosporidium* spp. (1.01%, 5/493) were also detected. For *G. intestinalis*, the highest environmental contamination rate was found in the community in western Mississippi (4.17%, 4/96). In contrast, two positive samples each were found in southwestern South Carolina (1.82%, 2/111), and one positive sample was found in southwestern Alabama (0.94%, 1/106). However, for *Cryptosporidium* spp., southwestern Alabama had the highest environmental contamination rate (3.92%, 4/102), and one additional positive soil sample was collected from south Texas (0.99%, 1/101). The only sample positive for *Entamoeba histolytica* was collected in southwestern Alabama (0.95%, 1/105).

### Relation of soil contamination of parasites to poverty.

**Figure 2.**
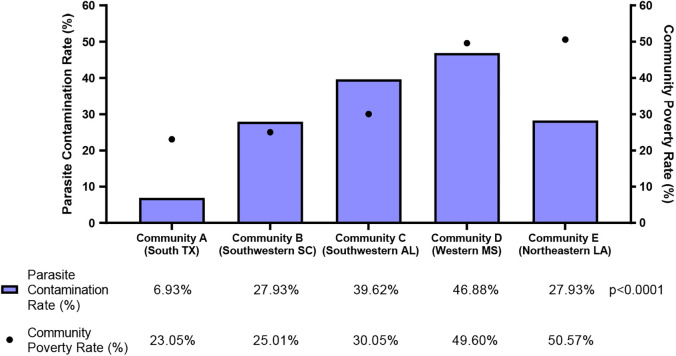
Overall parasite contamination rate (%, blue bars) and poverty rates (%, black dots) by community. There was a significant difference in the contamination rate between the communities (*P* <0.0001). The contamination rate was calculated as number of positive samples/total number of samples tested × 100%. Samples for any parasite tested were considered positive for the overall parasite contamination rate. Community poverty rates were obtained from the 2019 American Community Survey by the U.S. Census Bureau.

To characterize the relation between the environmental contamination of parasites and community poverty rates, the overall parasite contamination rate for each community was calculated and considered as an index despite the biological differences between the different parasites. Positive samples for any parasite tested were considered positive for this index. The relationship between poverty and parasite contamination is shown in Figure 2. Furthermore, the community in western Mississippi, which had one of the highest poverty rates of the communities studied (49.60%), featured the highest overall parasite contamination rate (46.88%). The parasite contamination rate generally decreased as poverty rates decreased across the communities, where the communities with the lowest poverty rate—southwestern South Carolina (25.01%) and south Texas (23.05%)—also had the lowest overall parasite contamination rates of 27.93% and 6.93%, respectively. However, northeastern Louisiana proved an outlier for this trend, with the highest poverty rate (50.57%) but an overall parasite contamination rate of only 27.93%.

**Figure 3.**
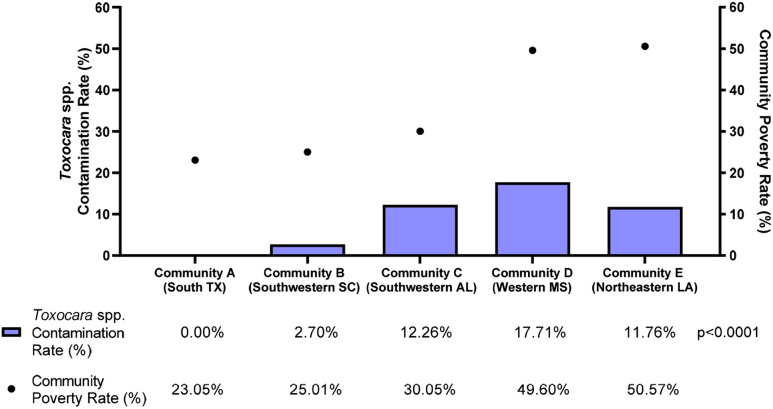
*Toxocara* spp. contamination rate (%, blue bars) and poverty rates (%, black dots) by community. There was a significant difference in the contamination rate between the communities (*P* <0.0001). The contamination rate was calculated as number of positive samples/total number of samples tested × 100%. Samples positive for *T. cati* or *T. canis* were considered positive for the *Toxocara* spp. contamination rate. Community poverty rates were obtained from the 2019 American Community Survey by the U.S. Census Bureau.

Examining specific parasites, the environmental contamination rates for *Toxocara* spp., which includes both *T. cati* and *T. canis*, displayed a similar relation to community poverty rates. The communities in the study had significantly different *Toxocara* spp. contamination rates (*P* <0.0001), and communities with higher poverty rates had higher contamination rates for *Toxocara* spp. (Figure 3). With the lowest poverty rate of 23.05%, south Texas had no soil samples positive for either *T. cati* or *T. canis*. Again, northeastern Louisiana was an outlier with the highest poverty rate (50.57%) but the third-highest *Toxocara* spp. contamination rate (11.76%). This relation between *Toxocara* spp. contamination rates and community poverty rates were further demonstrated by the positive correlation identified (*r*_s_ = 0.7000) (*P* = 0.233) (Figure not shown).

**Figure 4.**
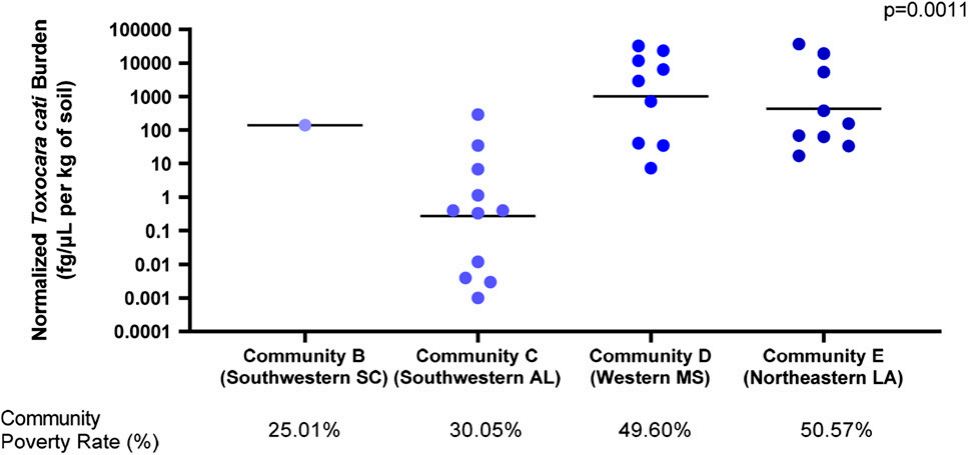
Normalized *Toxocara cati* burdens (fg/*µ*L of DNA per kg of soil) by the community. *T. cati* burdens were significantly greater for communities with higher poverty rates (*P* = 0.0011). Community poverty rates were obtained from the 2019 American Community Survey by the U.S. Census Bureau.

Regarding parasite burdens, quantified as the concentrations of the parasite DNA normalized to the mass of the soil sample, a significant difference was determined between *T. cati* burdens in the different communities in which it was detected (*P* = 0.0002) (Figure 4). Specifically, the median normalized burdens were 1,808 fg/µL of DNA per kg of soil and 155 fg/µL of DNA per kg of soil for western Mississippi and northeastern Louisiana, respectively—the two communities with the highest poverty rates of 49.60% and 50.57%, respectively. In contrast, southwestern Alabama, with a community poverty rate of 30.05%, had a median normalized *T. cati* burden of 0.362 fg/µL per kg of DNA.

The environmental contamination rates and burdens between public parks and private residences were also compared with examine one possible confounding factor for the relationship between poverty and parasites. However, the overall parasite contamination rate (*P* = 0.4583) and the contamination rate of *Toxocara* spp. (*P* = 0.8449) were not significantly different between public and private sampling sites. Furthermore, the burden of *T. cati* did not exhibit any significant difference (*P* = 0.8351) between public parks and private residences.

## DISCUSSION

### Environmental contamination of parasites

*Blastocystis* spp., the parasite with the highest environmental contamination rate (19.0%) in this study, is also the most common human parasite in the United States. Large-scale studies of the epidemiology of *Blastocystis* spp. in 2000 and 2004 identified the prevalence as 11% to 23% among the American population.^23^^,^^24^ Furthermore, using more sensitive molecular detection methods, the prevalence was as high as 62.8% in certain rural, low-income communities.^11^ However, no other studies of *Blastocystis* spp. in environmental samples in the United States exist. The environmental contamination rate of *Blastocystis* spp. could serve as an indicator of overall fecal–oral contamination in the environment.^19^^,^^24^

In contrast to *Blastocystis* spp., many studies have characterized the environmental presence of the other unicellular eukaryotic parasites detected in the study—*G. intestinalis* (1.40%) and *Cryptosporidium* spp. (1.01%). Studies of *G. intestinalis* and *Cryptosporidium* spp. in water have been conducted in low-income and high-income settings.^12^^,^^13^ Several such studies have characterized *Cryptosporidium* spp. or *G. intestinalis* contamination in wastewater or surface water in the United States.^25^^,^^26^ However, none of these studies examined soil contamination. Although *G. intestinalis* and *Cryptosporidium* spp. are primarily waterborne protozoa, they can also be found in the soil.^14^ Furthermore, Dai and Boll demonstrated that their ova attach to soil particles even in aquatic environments.^27^ Thus, soil may serve as an additional route of transmission for *G. intestinalis* and *Cryptosporidium* spp.

Of the helminths, *T. cati* (6.01%) and *T. canis* (3.61%) are the most prevalent pathogenic parasites found in this study. Toxocariasis, as both visceral larva migrans and ocular larva migrans, caused by either of these parasites is one of six Neglected Parasitic Infections in the United States designated by CDC.^28^ Recent NHANES surveys have shown varying prevalence from 5.1% to 13.9% among human populations.^29^ *Toxocara* spp. is also one of the few parasites with previous characterization in environmental studies.^15^^,^^16^ Domestic cats and dogs are the primary hosts of these parasites, therefore their detection in the soil in the communities may not indicate the prevalence in human populations.^28^ Nonetheless, environmental studies are critical for zoonotic pathogens because their life cycle requires maturation in the soil before transmission to humans.^30^

The soil-transmitted helminths *S. stercoralis* (2.00%), *T. trichiura* (1.80%), *A. duodenale* (1.42%), and *N. americanus* (0.20%), were also detected. These four parasites are the most common soil-transmitted helminths globally.^31^ In the United States, a previous large-scale study in Kentucky in 1982 found a prevalence of 12.6% for *T. trichiura*, 0.2% for *N. americanus*, and 3.0% for *S. stercoralis*.^32^ More recently, in a rural Alabama community, one similar to the communities selected for this study, the human infection prevalence was 34.5% for *N. americanus* and 7.3% for *S. stercoralis*.^10^ Although some studies have used soil samples for these helminths, none were conducted in the United States.^16^ Nonetheless, as soil-transmitted helminths, children can also ingest them from the soil. Furthermore, the burdens of these parasites in soil have been associated with their prevalence in their sampling areas. There is not always a direct correlation between parasitic human infection and soil contamination, likely because of exposure history, the life span of the parasites, and the location and climate of when the soil samples were collected.

Ultimately, this study is the first to examine soil samples for helminths and unicellular parasites in soils. The high contamination rates for *Blastocystis* spp. and *Toxocara* spp., as well as the detection of *Cryptosporidium* spp., *G. intestinalis*, *A. duodenale*, *T. trichiura*, *N. americanus*, and *S. stercoralis* in soils, may indicate their prevalence in their respective communities or serve as a source of infection.

### Relation of environmental contamination of parasites to poverty.

The significant associations between the contamination rates of parasites and community poverty rates further confirm the well-established relationship between the risk of contracting parasites and socioeconomic status.^4^ Doni et al. found that the poor socioeconomic status of families, in addition to children’s behavior playing with soil, had the most significant association with the risk of parasitic infections for a cohort of children in Turkey.^4^ However, our study does not elucidate the mediating factors in the relationship between parasites and poverty. One such mediating factor is likely poor sanitation. Rural communities in the United States frequently lack access to municipal sanitation systems and instead rely primarily on septic tanks, which require maintenance and are vulnerable to overflow and backup.^4^ The increased risk of exposure to raw sewage can also increase the risk of parasite infection.

*Toxocara* spp. is a zoonotic pathogen that cannot be transmitted person-to-person and is not associated with poor sanitation. However, the results demonstrated a significant association and a strong correlation between environmental contamination and community poverty rate. Similar results were identified in a study of soil samples from public parks in the boroughs of New York City by Tyungu et al.^33^ In that study, the percentage of parks positive for *Toxocara* spp. was significantly associated with the borough’s median income. Furthermore, the burdens of *Toxocara* spp. eggs differed significantly, with the highest burden in the borough with the lowest median income. These associations may be explained by the relationship between higher incomes and the ability to pay for veterinary checkups and deworming.^33^

Although our study demonstrated a relationship between parasites and poverty, it must be noted that the poverty rate is not the only factor that influences the environmental contamination rates of parasites. A major limitation of environmental sampling is the heterogeneity of the occurrence of parasites in samples.^34^ Furthermore, the contamination rates and burdens vary by soil type because sandy soils allow greater parasite burdens than clay or silt.^35^ Parasite burden is a potentially important factor: the more parasites detected in the soil, the higher the risk of human or animal infection, further increasing the life cycle of these parasites.^19^

### Limitations.

Although all attempts were made to maximize sample size per U.S. state, there were limitations on collecting and processing dirt samples. Ideally, locations for sample collection should include areas of higher incomes for better representation of the link between poverty and environmental parasites. Also, although the primer and probes sets (Table 2) are specific for parasite DNA sequences, there is cross-reactivity noted for the *Ancylostoma* primer/probe set that may detect the species *braziliense*, *caninum*, *ceylanicum*, and *tubaeforme* (https://blast.ncbi.nlm.nih.gov/Blast.cgi?PROGRAM=blastn&PAGE_TYPE=BlastSearch&LINK_LOC=blasthome). Several *Ancylostoma* spp. are zoonotic transmission but signify increased animal exposure, possibly associated with rural and poverty conditions. Future studies will include a species-specific primer/probe set for *A. duodenale.* The other parasite primer/probe sets were species-specific at the time of this study’s completion.

The communities in this study are also located in different regions of the United States with different climates. Moreover, the samples were collected across the five communities at various times of the year. Temperature, rainfall, and relative humidity have been shown to affect the incidence of *Cryptosporidium* spp., among other parasites, and seasonal variations in parasite infections such as *Cryptosporidium* spp. and *Blastocystis* spp. have been identified.^24^ Further work may attempt to elucidate the effects of some of these factors on parasite contamination rates and burdens, controlling for poverty rate. Alternatively, controlling for climatic factors may further elucidate the relationship between parasites and poverty rates.

## CONCLUSION

Several parasites were environmentally present in low-income communities in the southern United States. All communities in this study had high poverty rates, supporting the association between parasite contamination and poverty rates. This indicates greater parasite prevalence among human populations in communities with higher poverty rates and demonstrates a potential for transmission in these communities.
